# Advances in End-of-Life Care in Canada: Implications for Oncology Nursing

**DOI:** 10.3390/curroncol33010038

**Published:** 2026-01-09

**Authors:** Reanne Booker, Stephanie Lelond, Kalli Stilos

**Affiliations:** 1 Arthur JE Child Comprehensive Cancer Centre, 3395 Hospital Drive NW, Calgary, AB T2N 5G2, Canada; 2 Cancer Care Manitoba, 675 McDermot Avenue, Winnipeg, MB R3E 0V9, Canada; slelond2@cancercare.mb.ca; 3 Sunnybrook Health Science Centre, 2075 Bayview Avenue, Toronto, ON M4N 3M5, Canada; kalli.stilos@sunnybrook.ca

**Keywords:** oncology nursing, palliative care, medical assistance in dying, end-of-life care, advance care planning

## Abstract

This paper explores recent advancements in end-of-life (EOL) care in Canada, focusing on palliative care (PC) in oncology, advance care planning (ACP), and medical assistance in dying (MAiD). Despite improvements in cancer treatment, cancer remains a leading cause of death in Canada, with patients facing significant physical, psychosocial, and emotional challenges throughout the illness trajectory. Over the past few decades, PC has evolved to address serious illness from diagnosis onward, enhancing symptom management, quality of life, and patient satisfaction, while reducing hospital admissions and unnecessary treatments. However, barriers such as misconceptions about PC, late PC referrals, and limited access to PC, particularly in rural and remote areas, still exist. This perspective paper draws on the authors’ collective clinical and research experience in oncology and PC, complemented by a focused review of key literature. Ongoing education for oncology nurses on EOL care, including on PC, ACP, and MAiD, as well as continued efforts to expand access to PC for all Canadians, are imperative in order to improve the EOL experience for people affected by cancer nationwide.

## Simple Summary

In Canada, cancer remains a leading cause of death. Recent advances in end-of-life care have improved quality of life for patients. Palliative care, which focuses on relieving symptoms and supporting emotional, spiritual, and physical well-being, is now recommended from the time of diagnosis, alongside cancer treatment. Early integration of palliative care has been shown to reduce suffering, improve patient and caregiver quality of life, and sometimes even extend life after a cancer diagnosis. Advance care planning (ACP) helps patients express their values and make decisions about future care, and nurses play a key role in guiding these conversations. Medical assistance in dying (MAiD) is another option available to Canadians experiencing suffering after a cancer diagnosis. Ensuring oncology nurses have sufficient training, skills, and competencies in palliative care and advance care planning is essential for compassionate, patient-centered care throughout the illness trajectory, including at the end of life.

## 1. Introduction

Tremendous advances in the understanding of cancer biology, diagnostics, and treatment have led to improved outcomes for people with cancer, including better control of cancer and improvements in increased overall survival [1,2,3]. At the same time, cancer remains a leading cause of morbidity and mortality in Canada, with 239,100 Canadians diagnosed with cancer and 86,700 dying of cancer in 2023 [4]. Alongside such remarkable developments in oncology, there has also been notable progress in end-of-life care. This paper will provide an overview of the advances in EOL care in Canada over the past 10–15 years, including discussion of the integration of palliative care (PC) in oncology, advance care planning (ACP), and the option of medical assistance in dying (MAiD), and will provide an overview of implications for oncology nurses.

Cancer remains one of the leading causes of mortality in Canada, with more people being diagnosed each year [4]. While cancer survival rates have improved, they continue to vary widely depending on the type and stage of cancer at diagnosis, among other factors such as access to and equity of care [4,5,6]. In addition, research has found that patients with cancer experience an array of physical and psychosocial concerns throughout the illness trajectory, particularly in the year prior to death [7,8,9]. One of the most significant advances in EOL care in recent years has been the integration of PC in oncology [10,11,12]. As cancer treatment becomes increasingly more complex, with innovations such as immunotherapy and cellular therapies (such as chimeric antigen receptor T-cell therapy, bispecific antibodies, and antibody-drug conjugates), medical decision making for patients has also become more complex [13,14]. Such complexity, variability of prognoses, and the potential for high symptom burden underscore the need for integration of PC in oncology. This invited Perspective is informed by the authors’ clinical and research experience in oncology nursing and specialty PC and an appraisal of relevant peer-reviewed literature. Key clinical guidelines, consensus statements, and seminal studies related to EOL care in Canada were evaluated to contextualize current practice and highlight practical considerations. The aim is to review the state of EOL care in Canada, with attention to notable advancements such as the integration of PC in oncology, advance care planning, and medical assistance in dying. Current challenges and opportunities for further growth and development in EOL care will also be reviewed.

## 2. Evolution of Palliative Care

The World Health Organization (WHO) recognizes PC as a human right, describing it as appropriate from the time of diagnosis of a life-limiting illness, and alongside therapies such as chemotherapy and radiation therapy that are intended to prolong life [15]. In 2020, Radbruch et al. [16] established a new consensus-based definition of PC shifting from a disease-specific lens to serious health-related suffering and expanding its philosophy of compassionate care to integration within public health and health system frameworks, emphasizing equity, accessibility, and cultural responsiveness. Strengthening early and continuous integration across the health system, PC differentiates into primary or generalist vs. specialist PC, mandates evidence-informed practice, formalizes professional training expectations, and reframes PC as a human right and health system obligation, explicitly calling on governments to integrate PC into national health policies, all health services, insurance systems, and essential medicines lists.

Societal language around death and dying has utilized euphemisms for centuries, often rooted in fear of mortality and cultural taboos, sentimentalization, and the medicalization of death while removing it away from the home setting [17,18,19]. With many dying in the hospital setting among the general patient population, healthcare providers’ discomfort in facing death and caring for dying patients became a deficiency in care that could not be ignored. Balfour Mount [19] described the decreasing frequency of physician visits to patients’ rooms and decreasing nursing care provided as death becomes imminent, with a healthcare provider preference in treating those expected to recover. This lack of communication between providers, families, and patients fostered isolation, suspicion, and distrust, exacerbated by a reluctance to openly and honestly discuss diagnosis and prognosis. To better meet the needs of patients who were not expected to recover, a specialized PC unit with an interdisciplinary team was established alongside a palliative community-based service and a palliative consultative service. These services aimed to support quality of life, symptom control, and coping support.

Along with the evolving definition of PC, there has been a concurrent, albeit gradual shift in public and patient/caregiver perspectives on PC. For example, in 2016, Zimmermann et al. [20], conducted a qualitative study where patients with advanced cancer and their caregivers were interviewed about their attitudes and perceptions about PC. The authors found that participants held negative perceptions about PC, perceiving PC to have a “frightening association with death, hopelessness and dependency” ([20], p. E226). More recently, Chosich et al. [21] surveyed patients with cancer (N = 103) upon admission to an Australian tertiary cancer center. Of the 93% of patients who completed the questionnaire, 76% reported that they had heard of PC while 21% had received PC. Forty-five percent believed that PC was only associated with EOL and the majority (>80%) believed that they could receive both PC and cancer-directed treatment while receiving PC. Further, 77% reported that they felt comfortable with PC involvement. A minority of respondents reported feeling frightened (40%) or hopeless (29%) about a referral to PC. Of note, several authors have reported that once patients and family caregivers have heard an explanation of what PC is (and is not) and/or have experienced PC services firsthand, their perception of PC often shifts from negative to positive [20,22] and has been found to increase patient and caregiver hope [23].

Integration of PC in oncology began with the recognition in the 1990s and 2000s that cancer care must include symptom and quality-of-life support beyond curative intent. Over time, oncologists and PC teams began collaborative models of “simultaneous care” or “early PC” for patients with advanced cancer. In oncology, support for the early integration of PC concurrent with standard oncologic care has grown significantly in the past 15 years.

Strengthening access to PC has been identified as a key priority by Health Canada and requires collaboration among all health professionals, including nurses. In 2018, the Canadian government tabled the National Framework on Palliative Care in Canada that outlined strategies to help address access to and quality of PC in Canada [24]. In 2023, the Framework on Palliative Care in Canada—Five Years Later was published and reported that between 2016–2017 and 2021–2022, there was an increase in the number of Canadians who received some form of PC (58% compared to 52%) [25]. While this represents improvement, the report highlighted continued evidence of poor-quality PC, including late referrals with some people not receiving care until shortly before they died, and with most people still dying in hospital. For example, of people who received PC, 61% had received it in a hospital setting only, with 36% having received palliative home care [25]. In addition, access to specialty PC continues to be a challenge in rural and remote regions of Canada [26].

## 3. Benefits of Integration of Palliative Care in Oncology

**Figure 1:**
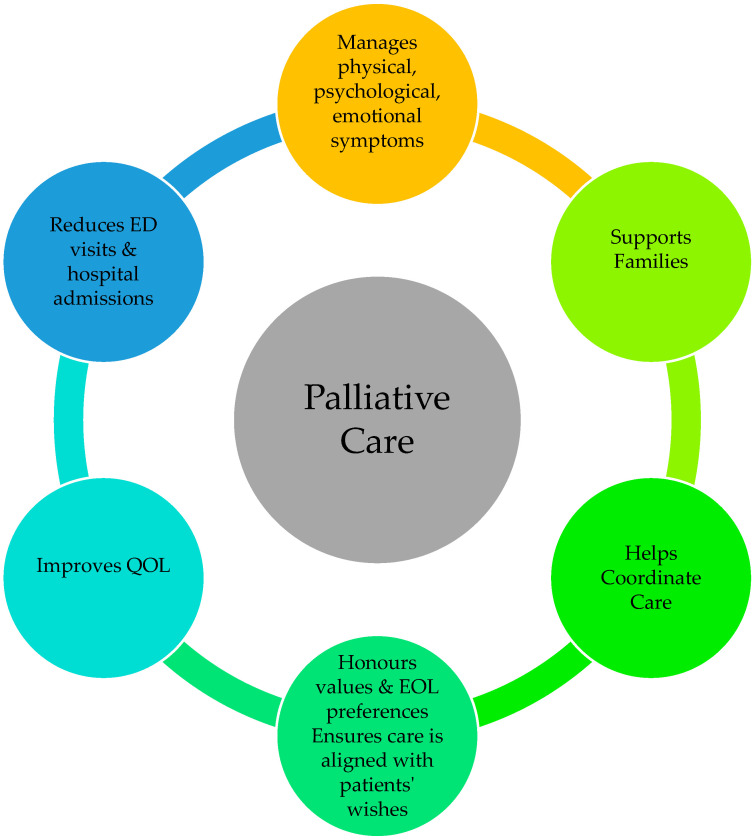
Benefits of palliative care. Note. EOL = end of life; QOL = quality of life; ED = emergency department.

Since the integration of concurrent palliative and oncologic care, the evidence base supporting this approach has expanded rapidly, beginning with the seminal study in advanced non–small cell lung cancer by Temel et al. [27]. Research now consistently demonstrates improved quality of life, better coordination of care, improved symptom management [28,29], and higher patient and caregiver satisfaction [30]. Furthermore, evidence supports its cost-effectiveness [31] and even suggests a potential survival benefit in some contexts [27,28]. An additional benefit includes improved illness and prognostic understanding, which facilitate medical decision making for patients and family caregivers/loved ones [32,33]. Early integration of PC has also been associated with reduction in healthcare utilization and aggressive interventions at EOL, including fewer emergency room visits and hospital admissions [34,35]. Recent systematic reviews and meta-analyses demonstrate consistent benefits of PC in oncology. In a meta-analysis of 19 studies, Cui et al. [36] reported that early PC was associated with improved quality of life (SMD = 0.14, 95% CI: 0.06–0.22) and reduced symptom burden (SMD = 0.14, 95% CI: 0.01–0.26). Similarly, Rogers et al. [11], in a critical appraisal of four randomized controlled trial (RCT) meta-analyses, found uniform evidence of quality-of-life improvement among patients receiving PC, with two analyses also identifying a survival benefit. Figure 1 summarizes these benefits of PC.

## 4. Barriers to Integration of Palliative Care in Oncology

Although there has been progress on societal perception of PC, myths and misperceptions remain a significant challenge to the integration of PC in oncology, harbored by patients [22,37]. Notably, many people continue to conflate PC with EOL or hospice care and consider that PC is something that can only be provided when there are no longer any cancer-directed treatments [38,39]. Associated with these misperceptions pertaining to PC are clinician concerns that discussing PC, particularly if viewed as the same as EOL or hospice care, may elicit fear or erode patients’ hope [40,41]. As such, clinicians may be reluctant to discuss PC with their patients to avoid causing any distress.

Lack of access to specialty PC providers is a significant barrier, particularly in rural and remote regions [42,43]. The absence of clear policies, guidelines, and/or protocols pertaining to PC referrals [43], increasing clinician workloads [44,45], and competing priorities [46] have all been reported as a challenge to PC integration in oncology. Finally, an additional clinician-related barrier is the lack of formal PC education and training clinicians receive [43,47,48].

## 5. Advance Care Planning

Advance care planning (ACP) ensures healthcare decisions align with patients’ values, particularly in critical or end-of-life situations. However, ACP should also address quality of life, the burdens patients are willing to bear, and their goals when facing serious illness. The two main goals of ACP are identifying a surrogate decision maker and establishing treatment preferences that reflect the patient’s wishes. Engaging patients and their substitute decision makers (SDMs) in ACP improves alignment between patient priorities and healthcare experiences, leading to more patient-centered care [49]. Engaging patients and their SDMs in ACP has been shown to yield multiple benefits, including better alignment between patients’ priorities and their healthcare experiences. Nurses are well positioned to bridge communication gaps and help ensure patients receive goal-concordant care. Traditionally, ACP discussions were primarily led by the patient’s most responsible physician; however, this practice has evolved to include other healthcare providers, including advanced practice nurses (APNs) such as nurse practitioners and clinical nurse specialists. The development of structured communication frameworks for clinicians, including nurses, has made it increasingly feasible for them to engage in ACP discussions with favorable outcomes. National guidelines, such as those from the Canadian Nurses Association [50], also support and outline the role of APNs in leading these critical conversations.

Despite its benefits, ACP often occurs too late. For example, Hasdianda et al. [51] found that only 37% of adults with serious illness had ACP discussions, typically just 33 days before death. In addition, other authors have reported that ACP discussions were often conducted by providers unfamiliar with the patient’s situation [52], and decision making often happened only after all treatment options were exhausted [53]. Nurses, who have sustained contact with patients and families, are well positioned to initiate ACP discussions earlier. Embedding these conversations into routine care, as recommended by Yarnell and Fowler [54], can make them more meaningful. Yet gaps remain. Cretu, Torabi, and Stilos [55] found APNs documented ACP discussions in only one-third of cases, highlighting the need to address barriers and better equip nurses to lead early ACP, ultimately improving patient and family care.

## 6. Medical Assistance in Dying (MAiD)

While PC endeavors to address all aspects of suffering, including physical, psychosocial, emotional, and spiritual/existential, it is recognized that some suffering may not be relieved by PC or may be refractory to available interventions. There is much that can be done to alleviate physical suffering, such as the use of various analgesics, nerve blocks, antiemetics, and an array of non-pharmacological interventions as well [56,57]. For refractory or intractable suffering, palliative sedation may be an option [58,59]. Some patients with cancer may also request information on, or request assessment for and provision of, a relatively new option available to Canadians, medical assistance in dying (MAiD).

**Figure 2:**
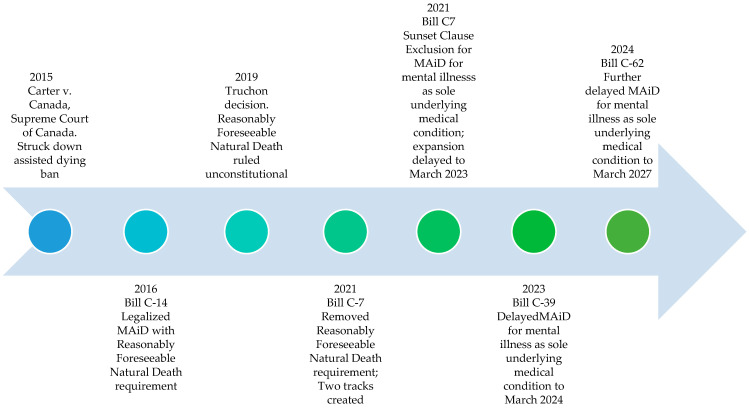
Timeline of key events related to legalization of MAiD in Canada. Note. MAiD = medical assistance in dying.

In 2016, changes to the Criminal Code of Canada meant that eligible individuals could request and receive MAiD [60,61,62]. Since then, there have been additional changes to the legislation, including amendments that came into effect in 2021 that created two tracks for people wishing to undergo MAiD (Table 1 and Figure 2). People whose natural deaths are considered reasonably foreseeable follow Track 1 and people whose natural deaths are not considered reasonably foreseeable follow Track 2 [60,61]. Safeguards have been established to protect vulnerable individuals and prevent abuse [60,61,62]; such safeguards are summarized in Table 2.

In the years since MAiD was legalized, many Canadian healthcare providers, including Nurse Practitioners, have had experience with MAiD, either as an assessor, a provider, or both [63]. Registered Nurses may also be involved in the care of patients who have requested or undergone MAiD, may serve as MAiD navigators/coordinators, or may also be involved in assisting with provision of MAiD, supporting patients and starting intravenous lines [64,65].

In 2023, 15,343 people died by MAiD, representing 4.7% of all Canadians who died in 2023 (up from 4.1% in 2022) [61]. Most MAiD provisions in 2023 were for people whose natural deaths were considered reasonably foreseeable (n = 14,721, 95.9%), congruent with 2021 and 2022 data. The median age of people who received MAiD under Track 1 was 77.7 and nearly 60% were over 75 years; 51.6% were men and 48.4% were women [61]. In 2023, cancer was the most frequently reported underlying medical condition for people receiving MAiD under Track 1, occurring in 64.1% of cases, consistent with previous years’ data [61]. Accordingly, oncology nurses are likely to encounter or may be involved in the care of patients who are wanting more information about MAiD, interested in pursuing it, or who request and receive MAiD.

The reasons people are requesting and receiving MAiD are collected by assessors/providers as part of the mandatory reporting requirements outlined by the federal government [62,66]. In 2023, the most common reason that people described as contributing to their suffering was the loss of the ability to engage in meaningful activities [61]. Additional issues that contributed to suffering for people in Track 1 included loss of ability to perform activities of daily living, loss of independence, and inadequate control of symptoms [61]. People who undergo MAiD assessments can report more than one source of suffering and most often report five sources of suffering [61]. It is important to note that the nature of suffering is reported by the practitioner conducting the assessment and is not directly reported by the person who requested MAiD [61,66].

In 2022, Liu et al. [67] reported on the experience of a Canadian academic hospital with MAiD between 2016–2020. The authors reported that 92 patients with cancer received MAiD during the study period and the leading cancer diagnoses were lung, colorectal, and pancreatic, congruent with national data [61,67]. Of note, 99% of the patients had distressing symptoms at time of their MAiD request; only one third of the patients with advanced/metastatic disease had received early PC [67]. The authors indicated that symptoms were considered distressing if the symptoms contributed to the patient’s request for MAiD and/or documentation in the patient’s chart prior to the MAiD assessment that noted distressing symptoms [67].

Russell et al. [8] examined symptoms experienced by patients with cancer during the last year of life and compared the symptom scores of those who underwent MAiD to those who did not undergo MAiD. The authors found that patients in both cohorts experienced worsening severity of symptoms in the year prior to death (β from 0.086 to 0.231, *p* ≤ 0.001 to 0.002). Patients who received MAiD had reported significantly greater anxiety (β = −0.831, *p* = 0.044) as well as greater lack of appetite (β = −0.934, *p* = 0.039) compared to those who had not received MAiD [8]. Most patients (65%) who received MAiD had submitted their request for MAiD within one month of their death. The findings suggest that patients with cancer who underwent MAiD did not experience higher symptom burden than patients with cancer who did not undergo MAiD [8]. Both studies [8,67] highlight the significance of attention to symptom assessment and management for patients with cancer and make the case for PC involvement. It is notable that one of the main reasons why a MAiD request was withdrawn in 2023 was that the individual felt that PC services were sufficient in relieving their suffering [61].

While the criminal law aspect of MAiD is regulated federally, the implementation and administration of MAiD are directed by provincial and territorial oversight, congruent with healthcare more broadly in Canada [62]. Accordingly, there may be variability in provincial MAiD programs and processes, provided that such programs and processes still abide by the federal legislation [64,65,68]. In addition, there may also be variability in terms of how PC and MAiD collaborate across Canada [69]. In some jurisdictions, MAiD and PC are closely aligned, with integrated programs, while in other areas MAiD and PC remain distinct and separate from one another [70,71,72,73].

Ethical considerations at the intersection of PC and MAiD include concerns about autonomy, protection of vulnerable populations, and equitable access to care, including access to both PC and MAiD [74,75,76]. Tensions arise between respecting autonomy and protecting persons from harm in a context of unequal social conditions and inequitable access to PC, disability supports, and other care that may help alleviate suffering [68,74,75,76]. The ethical defensibility of MAiD is contingent upon individual patient choice and on the justice and adequacy of the systems surrounding the individual’s choice. An individual’s choice to undergo MAiD must always be truly free and voluntary and not due to external pressures and structural coercion. Involving specialty PC can help with coordination of care and ensuring patients have access to supports and resources that can help assist with various aspects of an individual’s suffering [71,74].

It is important to acknowledge that there has been some tension around the legalization and expansion of MAiD. However, what is not contentious is the notion that all patients should have access to comprehensive PC and that lack of availability of or access to quality medical care and support should never be a reason that compels someone to request MAiD [77]. MAiD should not ever be seen as an easier or less expensive alternative to quality, comprehensive care that all Canadians deserve.

## 7. Implications for the Oncology Nurse

Given that the need for PC is expected to increase, coupled with a limited specialty PC workforce, there is a need for oncology nurses to possess primary PC skills and competencies [78]. In 2021, The Canadian Partnership Against Cancer (CPAC) and Health Canada published a framework on interdisciplinary PC competencies for healthcare providers, including nurses [79]. The Framework describes 12 domains of practice, with one domain being “principles of a palliative approach to care” ([79], p. 8). In addition, the framework outlines competencies for both generalist and specialist healthcare providers. In 2015, the Canadian Nurses Association, the Canadian Hospice Palliative Care Association, and the Canadian Hospice Palliative Care Nurses Group issued a joint position statement asserting that all nurses have a fundamental role in providing a palliative approach to care [80].

Oncology nurses play a crucial role in overcoming the barriers to integrating PC in oncology, such as addressing misconceptions about its role through public and professional education, advocating for earlier referrals, and improving access in rural areas. To improve the integration of PC palliative care in oncology, there is an urgent need for ongoing education and training for oncology nurses, particularly in areas of assessing and addressing symptom management, and ACP [78,81]. Oncology nurses are ideally positioned to initiate ACP discussions and ensure that care aligns with patients’ values and preferences [82]. This role has become even more critical with the legalization of medical assistance in dying (MAiD), which provides patients with another option for those experiencing refractory suffering. As more patients inquire about or request MAiD, oncology nurses will need to develop the competencies to navigate these complex ethical and clinical conversations [64,65]. The Hospice and Palliative Care Nurses’ Association suggests formal actions the oncology nurse can take when a patient asks about MAiD, including (1) clarifying the request, (2) evaluating and referring the patient for any unmanaged symptoms, (3) developing short-term and long-term plans with the patient to discuss their needs, and (4) collaborating with the interprofessional team to provide comprehensive care for the patient’s quality of life [83]. Ultimately, enhancing oncology nurses’ capacity to deliver primary PC, and skillfully navigating complex discussions such as those related to MAiD, will be instrumental in improving patient-centered outcomes and ensuring that care remains consistent with patients’ goals, needs, and preferences.

## 8. Conclusions

In Canada, advances in cancer treatment and EOL care, particularly the integration of PC in oncology, have significantly improved patient outcomes and quality of life. A key development in the Canadian healthcare landscape is the MAiD legislation, which intersects with PC. Despite these advances, cancer remains a leading cause of morbidity and mortality, and the increasing complexity of treatment options highlights the need for early and ongoing PC integration. Evidence consistently shows that early PC improves symptom management, enhances quality of life, and supports patients and caregivers in making informed decisions. Nurses play a critical role in providing compassionate care to patients considering MAiD, offering support in both symptom management and ethical decision making. Enhanced training for nurses in these areas will help ensure that patients receive the most comprehensive and respectful care possible.

## Figures and Tables

**Table 1:** Summary of legislation and court cases related to legalization of MAiD.

Year	Legislation/Case	Key Provisions
2015	Carter v. Canada (Supreme Court of Canada)	Ruling that prohibition on assisted dying violated the Canadian Chart of Rights and Freedoms for competent adults
2016	Bill C-14 NAME	Legalized MAiD for adults with serious and incurable illness, irreversible decline, intolerable suffering. Reasonably foreseeable natural death.
2019	Truchon v. Canada	Challenged Reasonably Foreseeable Natural Death requirement as being unconstitutional
2021	Bill C-7	Removed Reasonably Foreseeable Natural Death requirement; created two tracks: Track 1: death reasonably foreseeable Track 2: death not foreseeable (extra safeguards) Note: Mental illness as a sole underlying condition excluded
2021	Bill C-7 Sunset Clause	Added a 2-year sunset clause to allow MAiD for mental illness as sole underlying medical condition
2023	Bill C-39	Delayed eligibility for MAiD for mental illness as sole underlying medical condition to March 2024
2024	Bill C-62	MAiD for mental illness as sole underlying medical condition further delayed to 17 March 2027
Ongoing	Criminal Code of Canada	Federal regulations pertaining to eligibility criteria, safeguards, reporting requirements, practitioner protections

Note. MAiD = medical assistance in dying.

**Table 2:** Safeguards for Track 1 and Track 2 MAiD.

Safeguard	Track 1	Track 2
Eligibility	Death is reasonably foreseeable	Death is not reasonably foreseeable
Number of assessments	2 independent assessments (NP of physician)	2 independent assessments (NP of physician)
Underlying medical condition	Serious and incurable illness or disability	Serious and incurable illness or disability
State of decline	Advanced and irreversible decline in capability	Advanced and irreversible decline in capability
Nature of suffering	Irremediable suffering that is intolerable to the individual	Irremediable suffering that is intolerable to the individual
Expertise of assessors	No specific specialty expertise required for assessments	One assessor must have expertise in underlying medical condition or, must consult practitioner with expertise
Reflection period	No mandatory minimum reflection period	Minimum 90-day assessment period *
Informed consent	Must have capacity at time of eligibility assessment. If Waiver of Final Consent completed, informed consent must be given at time Waiver of Final Consent is signed	Required
Capacity at provision	Not required if Waiver of Final Consent has been completed	Required
Alternatives to MAiD	Must be informed of available options to relieve suffering	Must be informed of, and individual must seriously consider, alternatives to MAiD
Mental Illness as Sole Underlying Medical Condition	Excluded	Excluded until 17 March 2027
Oversight and Reporting	Must adhere to federal monitoring and reporting (Link/ref)	Must adhere to federal monitoring and reporting (Link/ref
Ethical Considerations	EOL choice, autonomy, beneficence (relief of suffering)	Concerns pertaining to vulnerability; voluntariness (if lack of supports/insufficient supports); protection against structural or social coercion

Note. MAiD = medical assistance in dying. NP = nurse practitioner. * may be shortened if death becomes reasonably foreseeable.

## Data Availability

No new data were created or analyzed in this study.

## Abbreviations

The following abbreviations are used in this manuscript:

ACP	Advance care planning
APN	Advanced practice nurse
EOL	End-of-life
PC	Palliative care
MAiD	Medical assistance in dying
